# The *18S* rRNA genes of *Haemoproteus* (Haemosporida, Apicomplexa) parasites from European songbirds with remarks on improved parasite diagnostics

**DOI:** 10.1186/s12936-023-04661-9

**Published:** 2023-08-10

**Authors:** Josef Harl, Tanja Himmel, Mikas Ilgūnas, Gediminas Valkiūnas, Herbert Weissenböck

**Affiliations:** 1 Department of Pathobiology, Institute of Pathology, University of Veterinary Medicine Vienna, Vienna, Austria; 2 Nature Research Centre, Vilnius, Lithuania

**Keywords:** Birth-and-death evolution, Semi-concerted evolution, *Parahaemoproteus*, Ribosomal genes

## Abstract

**Background:**

The nuclear ribosomal RNA genes of *Plasmodium* parasites are assumed to evolve according to a birth-and-death model with new variants originating by duplication and others becoming deleted. For some *Plasmodium* species, it has been shown that distinct variants of the *18S* rRNA genes are expressed differentially in vertebrate hosts and mosquito vectors. The central aim was to evaluate whether avian haemosporidian parasites of the genus *Haemoproteus* also have substantially distinct *18S* variants, focusing on lineages belonging to the *Haemoproteus majoris* and *Haemoproteus belopolskyi* species groups.

**Methods:**

The almost complete *18S* rRNA genes of 19 *Haemoproteus* lineages of the subgenus *Parahaemoproteus*, which are common in passeriform birds from the Palaearctic, were sequenced. The PCR products of 20 blood and tissue samples containing 19 parasite lineages were subjected to molecular cloning, and ten clones in mean were sequenced each. The sequence features were analysed and phylogenetic trees were calculated, including sequence data published previously from eight additional *Parahaemoproteus* lineages. The geographic and host distribution of all 27 lineages was visualised as *CytB* haplotype networks and pie charts. Based on the *18S* sequence data, species-specific oligonucleotide probes were designed to target the parasites in host tissue by in situ hybridization assays.

**Results:**

Most *Haemoproteus* lineages had two or more variants of the *18S* gene like many *Plasmodium* species, but the maximum distances between variants were generally lower. Moreover, unlike in most mammalian and avian *Plasmodium* species, the *18S* sequences of all but one parasite lineage clustered into reciprocally monophyletic clades. Considerably distinct *18S* clusters were only found in *Haemoproteus tartakovskyi* hSISKIN1 and *Haemoproteus* sp. hROFI1. The presence of chimeric *18S* variants in some *Haemoproteus* lineages indicates that their ribosomal units rather evolve in a semi-concerted fashion than according to a strict model of birth-and-death evolution.

**Conclusions:**

Parasites of the subgenus *Parahaemoproteus* contain distinct *18S* variants, but the intraspecific variability is lower than in most mammalian and avian *Plasmodium* species. The new *18S* data provides a basis for more thorough investigations on the development of *Haemoproteus* parasites in host tissue using in situ hybridization techniques targeting specific parasite lineages.

**Supplementary Information:**

The online version contains supplementary material available at 10.1186/s12936-023-04661-9.

## Background

The ribosomal RNAs constitute the core part of the ribosomes, which are essential for protein synthesis in all cells. The nuclear ribosomal units of eukaryotes contain the genes for *18S* rRNA, *5.8S* rRNA, and *28S* rRNA, separated by the internal transcribed spacers ITS1 and ITS2. The *5S* rRNA genes are in different genomic regions. The *18S* rRNA is part of the small ribosomal subunit (SSU), and *28S* rRNA, *5.8S* rRNA, and *5S* rRNA are part of the large ribosomal subunit (LSU). The ribosomal units of most eukaryotes are arranged in clusters of tandem repeats on one or several chromosomes, whereby each cluster contains multiple copies of ribosomal units. Due to functional constraints, the nuclear ribosomal RNAs are among the most conserved genes in eukaryotes. Ribosomal units are assumed to evolve according to a model of concerted evolution that leads to homogenization [1, 2]. Mechanisms of concerted evolution likely involve unequal crossing over during recombination, gene duplication, and inter-chromosomal gene conversion [3]. However, the nuclear ribosomal genes of *Plasmodium* parasites are exceptional because their ribosomal units are assumed to evolve according to a birth-and-death model with new variants originating by duplication and others becoming deleted [4]. Studies on the *18S* rRNA genes of human and rodent *Plasmodium* species found that the sequences of individual units can vary substantially [5], and distinct variants are expressed differentially in the vertebrate and mosquito hosts [6]. The *18S* sequences expressed in the vertebrate hosts and mosquito vectors were named A-type and S-type variants, respectively [6, 7]. This pattern was found in rodent, simian, and human *Plasmodium* species (except for *Plasmodium malariae*), with A-type and S-type differing by 10% to 17% [8].

The first comprehensive study on nuclear ribosomal genes of avian haemosporidian parasites was published by Harl et al*.* [8], who sequenced the *18S* genes of seven *Plasmodium*, nine *Haemoproteus*, and 16 *Leucocytozoon* lineages. Most avian *Plasmodium* lineages studied also feature clusters of distinct *18S* variants, differing by up to 14.9%. A similar pattern was found in the *Leucocytozoon toddi* group, but the *18S* sequences of other *Leucocytozoon* and *Haemoproteus* parasites were less variable and lacked highly diverged clusters of variants, except for *Haemoproteus tartakovskyi* [8].

The genus *Haemoproteus* currently comprises about 180 morphologically described species, but molecular genetic data is available from less than half of the species [9]. The MalAvi database (http://130.235.244.92/Malavi/; accessed in December 2022) contains more than 1500 unique *Haemoproteus* lineages covering the entire or almost entire 478 bp *CytB* barcode region. The vast majority has not been linked to morphospecies yet, therefore, the known species only constitute a fraction of the species diversity. The genus includes the two subgenera *Haemoproteus* and *Parahaemoproteus*. Parasites of the subgenus *Haemoproteus* are mainly found in birds of the orders Columbiformes, Suliformes, and Charadriiformes and are transmitted by louse flies (Hippoboscidae). Parasites of the subgenus *Parahaemoproteus* are extremely diverse in passeriform birds globally, particularly in the northern hemisphere, and are transmitted by biting midges (Ceratopogonidae).

For the present study, the *18S* rRNA genes of 19 *Haemoproteus* lineages were sequenced. About half of the investigated lineages are currently linked to the *Haemoproteus majoris* (hCCF5, hCWT4, hEMSPO03, hPARUS1, hPHSIB1, and hWW2) and *Haemoproteus belopolskyi* (hACDUM2, hARW1, hMW3, and hSW1) groups, which include some of the most common avian haemosporidian lineages in Palearctic passeriform birds. Moreover, the *18S* sequences were sequenced of *Haemoproteus balmorali* hCOLL3, *Haemoproteus fringillae* hCCF3, *Haemoproteus* sp. hROFI1, *Haemoproteus nucleocondensus* hGRW01, *Haemoproteus parabelopolskyi* hSYAT02, *Haemoproteus payevskyi* hRW1, *Haemoproteus* cf. *magnus* hCCF6, and the yet unlinked lineages hCCF2 and hCWT7. The sequences of eight *Haemoproteus* lineages previously published by Harl et al*.* [8] were included in the analyses.

The main question was whether avian *Haemoproteus* parasites feature clusters of distinct *18S* variants like many *Plasmodium* species, which would indicate that their ribosomal genes could also be differentially expressed in the bird hosts and dipteran vectors. Moreover, the ribosomal genes constitute a large part of the RNA molecules in cells and therefore are suitable targets for molecular genetic approaches such as in situ hybridization assays. The *18S* sequences were the basis for designing species/lineage-specific oligonucleotide probes, which can be used to target parasites in histological sections and differentiate parasites in co-infections.

## Methods

### Sample collection and preparation

For the present study, the *18S* rRNA genes of 19 *Haemoproteus* lineages found in passeriform birds in the Palearctic were sequenced. The samples were part of the collections of the Nature Research Centre in Vilnius (Lithuania) and the Institute of Pathology at the University of Veterinary Medicine Vienna (Austria). Wild birds were collected at the Ornithological Station in Ventė Cape (Lithuania) using stationary traps (large ‘Rybachy’ type, zigzag and funnel traps) and mist nets between 2018 and 2021, and blood samples were taken with heparinised microcapillaries after puncturing the brachial vein. Drops of fresh blood were used to prepare blood spots on filter paper for DNA analysis and several blood films on glass slides for microscopic examination. The blood films were fixed in absolute methanol for one minute and then stained with 10% Giemsa [10]. The blood films were analysed by microscopic examination and 60 birds with high parasitaemia were euthanised by decapitation according to permits (see Ethical statement). Blood samples were taken from two birds (AH1663 and AH1664) during routine bird ringing at the Biological Station Neusiedler See (Illmitz, Burgenland) in 2018 as described above. Tissue samples (liver) were taken from one dead bird (AH2023) submitted to the Institute of Pathology (Vetmeduni Vienna) for a citizen science study in 2020 [11] and stored at minus 80 °C for DNA analysis. Organ samples of the dead birds were fixed in formalin and embedded in paraffin (FFPE) and deposited in the tissue collection of the Institute of Pathology (Vetmeduni Vienna). Voucher blood films of the Lithuanian samples were deposited at the Nature Research Centre. DNA was isolated either from blood spots on filter papers or frozen liver tissue using the DNeasy Blood & Tissue Kit (QIAGEN, Venlo, Netherlands). The manufacturer’s protocol was followed for isolation of DNA from tissue but two eluates of 100 µl each were made from the same column, the first at 8000 rpm, and the second at 13,000 rpm. The second eluate was used for the PCRs. Information on the samples analysed for the present study is provided in Table 1. The table also includes information on the samples studied by [8], who previously published *18S* sequences of eight *Parahaemoproteus* lineages.

**Table 1:** Samples analysed in the present study

Sample	*Species*	Lineage	Host species	Country	ID LT
AH0004H	*H. attenuatus*	hROBIN1	*Luscinia luscinia*	RU	298/14c
**AH1982H**	*H. balmorali*	hCOLL3	*Ficedula hypoleuca*	LT	H41/19R
**AH1896H**	*H. belopolskyi*	hACDUM2	*Acrocephalus scirpaceus*	LT	159/18R
**AH1899H**	*H. belopolskyi*	hMW3	*Acrocephalus palustris*	LT	187/18R
**AH1902H**	*H. belopolskyi*	hMW3	*Acrocephalus palustris*	LT	203/18R
**AH1664H**	*H. belopolskyi*	hSW1, (hSW3 uncertain*)	*Acrocephalus schoenobaenus*	AT	V034148
**AH1903H**	*H. belopolskyi*	hARW1	*Acrocephalus palustris*	LT	206/18R
AH0460H	*H. brachiatus*	hLK03	*Falco tinnunculus*	AT	–
AH0608H	*H. dumbbellus*	hEMCIR01	*Emberiza citrinella*	AT	–
**AH1973H**	*H. fringillae*	hCCF3, hCCF5*, hROFI1*	*Fringilla coelebs*	LT	H19/19R
**AH2168H**	*H. fringillae*	hCCF3, hCCF3*, hCCF5*	*Fringilla coelebs*	LT	H06/21R
AH0002H	*H. lanii*	hRB1	*Lanius collurio*	RU	147/14c
**AH2153H**	*H.* cf. *magnus*	hCCF6	*Fringilla coelebs*	LT	H20/20R
**AH2154H**	*H.* cf. *magnus*	hCCF6	*Fringilla coelebs*	LT	H27/20R
**AH1973H**	*H. majoris*	hCCF5, hCCF3*, hROFI1*	*Fringilla coelebs*	LT	H19/19R
**AH1981H**	*H. majoris*	hCWT4	*Sylvia curruca*	LT	H40/19R
**AH2023H**	*H.* cf. *majoris*	hEMSPO03	*Pyrrhula pyrrhula*	AT	–
**AH2163H**	*H. majoris*	hPARUS1	*Cyanistes caeruleus*	LT	73/15c
**AH1977H**	*H. majoris*	hPHSIB1	*Phoenicurus ochruros*	LT	H31/19R
**AH1893H**	*H. majoris*	hWW2	*Sylvia atricapilla*	LT	98/18R
**AH2171H**	*Haemoproteus* sp*.*	hROFI1, hCCF6*	*Fringilla coelebs*	LT	H41/21R
AH0014H	*H. minutus*	hTURDUS2	*Turdus merula*	RU	46/14c
**AH1663H**	*H. nucleocondensus*	hGRW01	*Acrocephalus arundinaceus*	AT	–
**AH1895H**	*H. parabelopolskyi*	hSYAT02	*Sylvia atricapilla*	LT	137/18R
**AH1887H**	*H. payevskyi*	hRW1	*Acrocephalus scirpaceus*	LT	13/18R
**AH2151H**	*Haemoproteus* sp.	hCCF2	*Fringilla coelebs*	LT	H12/20R
**AH1974H**	*Haemoproteus* sp.	hCWT7	*Sylvia communis*	LT	H27/19R
AH0775H	*H. syrnii*	hCULKIB01	*Strix uralensis*	AT	–
AH0141H	*H. syrnii*	hSTAL2	*Strix uralensis*	AT	–
AH0776H	*H. syrnii*	hSTAL2	*Strix uralensis*	AT	–
AH0005H	*H. tartakovskyi*	hSISKIN1	*Loxia curvirostra*	RU	399/14c

Individual IDs, parasite species, MalAvi lineage, bird species, country, IDs of the Nature Research Centre (Vilnius, Lithuania). Asterisks indicate other lineages contained in co-infection. Samples with IDs in bold letters were first analysed in the present study, the others were analysed previously [8]. The country codes stand for Austria (AT), Lithuania (LT), and Russia (Kaliningrad Oblast, RU)

### *CytB* PCR primers

The nested PCR assay described by [12] was used to obtain a 478 bp fragment of the *Cytochrome B* (*CytB*) gene, the common DNA barcode sequence for avian haemosporidian parasites. PCRs were performed using the primers HaemNFI (5′-CAT ATA TTA AGA GAA NTA TGG AG-3′) and HaemNR3 (5′-ATA GAA AGA TAA GAA ATA CCA TTC-3′) in the first PCR, and HaemF (5′-ATG GTG CTT TCG ATA TAT GCA TG-3′)/HaemR2 (5′-GCA TTA TCT GGA TGT GAT AAT GGT-3′) and HaemFL (5′-ATG GTG TTT TAG ATA CTT ACA TT-3′)/HaemR2L (5′-CAT TAT CTG GAT GAG ATA ATG GNG C-3′) in the nested PCRs, respectively [12]. The primers CytB_HPL_intF1 (5′-GAG AAT TAT GGA GTG GAT GGT G-3′) and CytB_HPL_intR1 (5′-ATG TTT GCT TGG GAG CTG TAA TC-3′) [8] were used to obtain 885 bp sections of the *CytB*, which were used to calculate the phylogenetic trees.

### *18S* PCR primers

To amplify the almost entire *18S* rRNA genes of the *Haemoproteus* lineages, the primers 18S_H_1F (5′-TGG TTG ATC TTG CCA GTA ATA TAT GT-3′) and 18S_H_1R (5′-CGG AAA CCT TGT TAC GAC TTTTG-3′) were used, which are located at the 5′-end and 22 bp from the 3′-end of the *18S*, respectively [8]. Since the *18S* sequence reads of some *Haemoproteus* lineages did not overlap, the *Haemoproteus*-specific primers 18S_H_int_F (5′-AGA TCA AGT TGA AGT GCC AGC ATT-3′) and 18S_H_int_R (5′-CGT TAA ACA CGC GAC GTC-3′) were designed, which are located approximately 550 bp and 1700 bp from the 5′-end of the *18S*, to sequence a 1100 bp section covering the middle part. The latter primers only target the *18S* rRNA genes of the subgenus *Parahaemoproteus*, but not those of the genetically highly diverged subgenus *Haemoproteus* [8].

### PCRs and molecular cloning

The PCRs targeting the 478 bp *CytB* barcode section followed the protocol of [12] and were performed using the GoTaq® G2 Flexi DNA Polymerase (Promega, Wisconsin, Madison, USA). The PCRs started with an initial denaturation for 2 min at 94 °C, followed by 35 cycles with 30 s at 94 °C, 30 s at 50 °C, 1 min at 72 °C, and a final extension for 10 min at 72 °C. Each 1 µl of the first PCR-product was used as template in the two nested PCRs. The PCRs targeting the 885 bp *CytB* fragment were performed with the GoTaq® Long PCR Master Mix (Promega, Wisconsin, Madison, USA). The PCRs started with an initial denaturation for 2 min at 94 °C, followed by 35 cycles with 30 s at 94 °C, 30 s at 55 °C, 2 min at 68 °C, and a final extension for 10 min at 72 °C. The PCRs targeting the *18S* sequences were performed with the GoTaq® Long PCR Master Mix under the same conditions but with 2 min extension time. Each two PCRs were done to obtain sufficient PCR product for direct sequencing and molecular cloning. The PCR products were visualised on 1% LB agarose gels and sent to Microsynth Austria GmbH (Vienna, Austria) for purification and sequencing in both directions using the PCR primers.

The *18S* PCR products were then further processed and subjected to molecular cloning as described in [8]. Each 20 µl of PCR-product were run on 1% LB agarose gels and the bands were excised with flamed spatulas. The gel bands were purified using the QIAquick Gel-Extraction Kit (QIAGEN) following the standard protocol and eluted with 20 µl distilled water. Cloning was performed with the TOPO™ TA Cloning™ Kit (Invitrogen, Carlsbad, California, USA) using the pCR™4-TOPO® vector and One Shot® TOP10 competent cells. After ligation and transformation, the *Escherichia coli* cells were recovered in SOC medium for 1 h at 37 °C, plated on LB agar plates, and grown for 20 h at 37 °C. From each cloning assay, 15 to 20 individual clones were picked with sterilised (flamed) tooth sticks and transferred to fresh LB agar plates. The same tooth sticks with remaining *E. coli* were twisted in PCR-tubes with 25 µl master mix for the colony-PCRs. Colony-PCRs were performed with the GoTaq® Long PCR Master Mix (Promega) under the same conditions as the *18S* PCRs (see above) but using the primers M13nF (5′-TGT AAA ACG ACG GCC AGT GA-3′) and M13nR (5′-GAC CAT GAT TAC GCC AAG CTC-3′) [8]. The PCR-products of up to 15 clones carrying inserts of the expected size were sent to Microsynth Austria GmbH (Vienna, Austria) for purification and sequencing using the colony-PCR primers.

### Analysis of raw sequence data

The forward and reverse reads and electropherograms of the *CytB* and *18S* sequences were aligned manually and checked by eye in BioEdit v.7.0.8.0 [13]. Then the sequences were aligned and sorted with MAFFT v.7 [14] using the default option (FFT-NS-2), and primer and cloning vector sequences were cut from the *18S* alignments. Since some *18S* clones did not overlap, the middle part was re-sequenced from 65 clones with the primers 18S_H_int_F and 18S_H_int_R. The long and the short *18S* sequences were combined and realigned with MAFFT v.7. and all aberrant positions were rechecked in the corresponding electropherograms.

### *CytB* haplotype networks

To visualise the geographic and host distribution of the *H. majoris* and *H. belopolskyi* lineages, two DNA haplotype networks were calculated. The *CytB* lineages of both groups cluster in reciprocally monophyletic clades, which currently contain 21 (*H. majoris*) and 40 (*H. belopolskyi*) lineages, most of which have not been linked to morphospecies yet. The clades were identified by calculating a Maximum Likelihood (ML) tree based on all complete *Haemoproteus* lineages listed in the MalAvi database (http://130.235.244.92/Malavi/index.html) and a few other lineages available from NCBI GenBank only. The sequences were aligned with MAFFT v.7. [14] applying the default option (FFT-NS-2) and the first and last two bp were trimmed because they were erroneous or incomplete in some sequences. A ML bootstrap tree (1000 replicates) was calculated with IQ-TREE v.1.6.12. [15] based on the trimmed 474 bp alignment (1325 unique lineages) applying the substitution model GTR+F+I+G4. For each lineage contained in the *H. majoris* and *H. belopolskyi* clades, the information on hosts, countries, and references was extracted from the MalAvi “Hosts and Sites” table (http://130.235.244.92/Malavi/) and organised in a Microsoft Excel (Microsoft, Redmond, WA, USA) sheet. Moreover, sequences and related information for some lineages, which were available on NCBI GenBank only, were added. The Median-Joining haplotype networks were calculated with Network 10.2.0.0 (Fluxus Technology Ltd, Suffolk, UK) using the default settings. The networks were graphically arranged and provided with information on hosts species/families and geographic regions according to the United Nations geoscheme with Network Publisher v.2.1.2.5 (Fluxus Technology Ltd). To show the geographic and host distribution of the other lineages, pie charts were created with Microsoft Excel based on the MalAvi “Hosts and Sites” data. All graphics were finalised with Adobe Illustrator CC v.2015 (Adobe Inc., San José, CA, USA).

### Phylogenetic trees

ML and Bayesian Inference (BI) trees were calculated for both the *CytB* and *18S* data sets. *CytB* trees were calculated based on the 885 bp alignment of the 19 MalAvi lineages obtained in the present study and eight lineages published by [8]. A sequence of *Plasmodium matutinum* pLINN1 (MT912161) was included as outgroup. The best fit substitution model suggested by IQ-TREE v.1.6.12. [15] according to the corrected Akaike Information Criterion (AICc) was GTR+F+I+G4. The ML bootstrap tree was calculated with IQ-TREE v.1.6.12. [15] by performing 10,000 bootstrap replicates. The BI tree was calculated with MrBayes v.3.2. [16]. The analysis was run for 5 million generations (2 runs each with 4 chains, one of which was heated) and every thousandth tree was sampled. The first 25% of trees were discarded as burn-in and a 50% majority rule consensus tree was calculated from the remaining 3750 trees.

The alignment of *18S* sequences contained 201 clones of 19 MalAvi lineages obtained in the present study and 71 clones of seven MalAvi lineages published by [8]. Thirteen clones were excluded from the analyses because they originated from other lineages present in co-infections. The *18S* sequences of the sample AH0002H (hRB1) from [8] were also excluded because they lacked a 115 bp section at the 5′-end. The sequences were aligned with MAFFT v.7. [14] using the option G-INS-I (globally alignment based on Needleman-Wunsch algorithm). To reduce the number of sequences, subsets of two to five distinct clones per MalAvi lineage (74 sequences in total) were selected. An outgroup was not included because the *18S* sequences of other haemosporidian genera differ strongly from those of *Parahaemoproteus* spp. and the removal of gap position would have led to the loss of information. The subset of sequences was realigned with MAFFT v.7. (G-INS-I option), resulting in a 2526 bp alignment. The first 65 and the last 18 positions were removed because they were not present in the sequences of samples AH1982 (hCOLL3) and AH1895 (hSYAT02) (obtained by direct sequencing), and in the sample AH0608 (hEMCIR01; [8]). After trimming the latter sites, the alignment featured 2443 positions. After removing all sites containing gaps with trimAl v.1.2. [17], the final alignment featured 1605 positions. The best fit substitution model suggested by IQ-TREE v.1.6.12. [15] according to the corrected Akaike Information Criterion (AICc) was TVM+F+I+G4. Since the latter model is not implemented in MrBayes v.3.2. [16], the second-best model GTR+F+I+G4 was used for both the ML and BI analyses. The ML bootstrap and the BI trees were calculated with IQ-TREE v.1.6.12. [15] and MrBayes v.3.2. [16] applying the same parameters as used for inferring the *CytB* trees.

### Sequence comparison and recombination tests

Prior to the analysis of sequences, the *18S* clones of each MalAvi lineage were placed in separate files to determine the minimum and maximum lengths of sequences. The mean GC-contents of *18S* sequences from each MalAvi lineage were calculated with Microsoft Excel. The sequences of each MalAvi lineage were aligned separately with MAFFT v.7. [14] using the option G-INS-I, and maximum *p*-distances between the variants were calculated with MEGA X v.10.0.5 [18]. The latter alignments were also used to test whether distinct *18S* clones from the same MalAvi lineages showed chimeric features, thus indicating recombination between different *18S* variants from the same MalAvi lineages. RDP5 v.5.3. [19]) was used to perform the following recombination tests: RDP [20], Bootscan [21], GENECONV [22], Maxchi [23], Chimaera [24], SiSscan [25], and 3Seq [26].

### Design of probes for in situ hybridization

Based on the alignments of all *Haemoproteus 18S* sequences, oligonucleotide probes were designed, which specifically target the investigated lineages. The alignment was inspected by eye to identify suitable regions for probe binding. Most probes were placed in variable sequence regions specific to each one MalAvi lineage. The quality of the probes was checked with AmplifX v.2.0.7 (Nicolas Jullien, Aix-Marseille Univ., CNRS, INP, Marseille, France; https://inp.univ-amu.fr/en/amplifx-manage-test-and-design-your-primers-for-pcr). All probes were blasted against genomes of apicomplexan parasites and birds in NCBI GenBank to exclude unintentional binding.

## Results

The *18S* sequences of 19 *Parahaemoproteus* lineages, half of which belong to the *H. majoris* and *H. belopolskyi* groups, were analysed. The *18S* sequences published for seven *Haemoproteus* lineages by [8] were included in the statistical analyses and phylogenetic trees.

### *CytB* haplotype networks

To visualise the geographic and host distribution of *CytB* lineages belonging to the *H. majoris* and *H. belopolskyi* groups, DNA haplotype networks were calculated based on 474 bp alignments containing data of all lineages clustering in two clades.

**Fig. 1:**
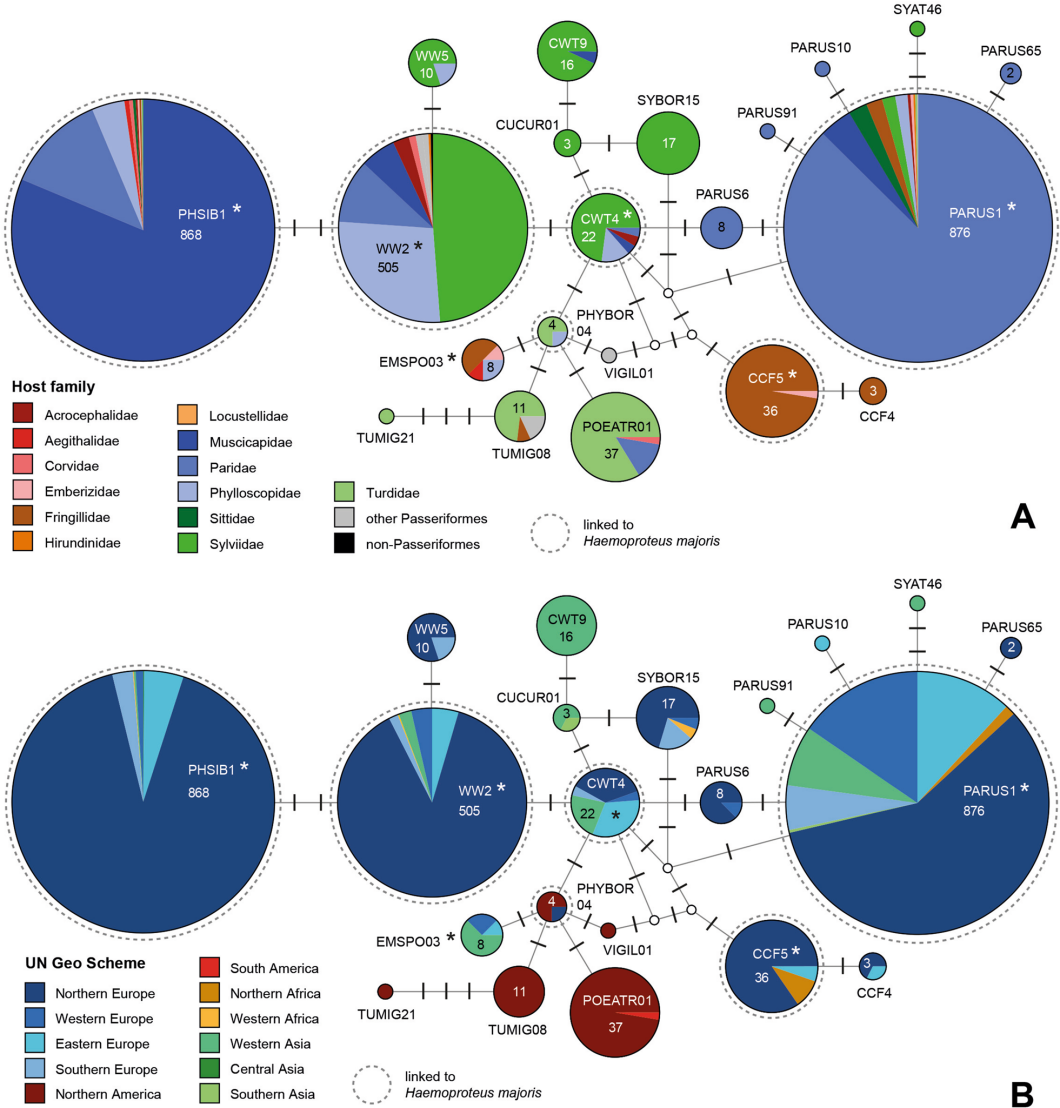
Median‑Joining DNA haplotype network of partial (474 bp) *CytB* sequences belonging to the *Haemoproteus majoris* group. The two figures show the distribution in **A** bird families and **B** geographic areas according to the United Nations geoscheme. Each circle represents a unique haplotype/lineage. The frequency is indicated for all haplotypes with more than one record and roughly corresponds to the size of circles. Bars on branches indicate the number of substitutions between two haplotypes. Small white circles represent median vectors, which are hypothetical (often ancestral or unsampled) sequences required to connect existing haplotypes with maximum parsimony. The lineages analysed in the present study are marked with asterisks

The *H. majoris* clade included 21 unique lineages differing by up to nine bp in the 474 bp *CytB* sequence (Fig. 1). Most lineages were predominantly found in songbirds in Europe and Western Asia, and occasionally in Northern Africa and Southern Asia, except for hPOEATR01, hTUMIG08, hTUMIG21, and hPHYBOR04, mainly from Northern American thrushes. According to the MalAvi database (http://130.235.244.92/Malavi/), the following six lineages were attributed to *H. majoris* by [27, 28]: hCCF5, hCWT4, hPARUS1, hPHSIB1, hPHYBOR04, and hWW2. The *18S* sequences were obtained from all the latter lineages except for hPHYBOR04, because the only sample available did not contain sufficient DNA. The three lineages hPARUS1, hPHSIB1, and hWW2 are among the most common haemosporidian parasite lineages in Eurasian songbirds. hPARUS1 (876 records) was mainly found in Northern Europe (510), Western Europe (135), Eastern Europe (104), Western Asia (65), and Southern Europe (48); the main hosts are *Cyanistes caeruleus* (463) and *Parus major* (244) of the family Paridae. hPHSIB1 (868) was mostly found in Northern Europe (791) and Eastern Europe (44); the Muscicapidae species *Ficedula albicollis* (499) and *Ficedula hypoleuca* (172) are the most common hosts. hWW2 (505) was mostly found in Northern Europe (445) and has a wider host spectrum with about half of the records from Sylviidae (247), followed by Phylloscopidae (138), Paridae (55), and Muscicapidae (31); the most common host species are *Phylloscopus trochilus* (108), *Sylvia borin* (90), *Sylvia atricapilla* (79), and *Sylvia communis* (64). hCCF5 (36) was almost exclusively found in *Fringilla coelebs* (35) in Northern Europe (32) and Northern Africa (4). hCWT4 (22) was mainly found in Northern Europe (8), Eastern Europe (7), and Western Asia (5), with most records from Sylviidae (15), particularly *S. communis* (8). hEMSPO03 (8) has not been linked to a morphospecies yet; it was found in Western Asia (5), Western Europe (2), and Eastern Europe (1) in *Pyrrhula pyrrhula* (3) and *Phylloscopus nitidus* (2) and others. Records of hEMSPO03 from *Phylloscopus humei* (1) and *Phylloscopus trochiloides* (3) in India [29] were not included because the sequences covered only 448 bp of the *CytB* barcode region.

**Fig. 2:**
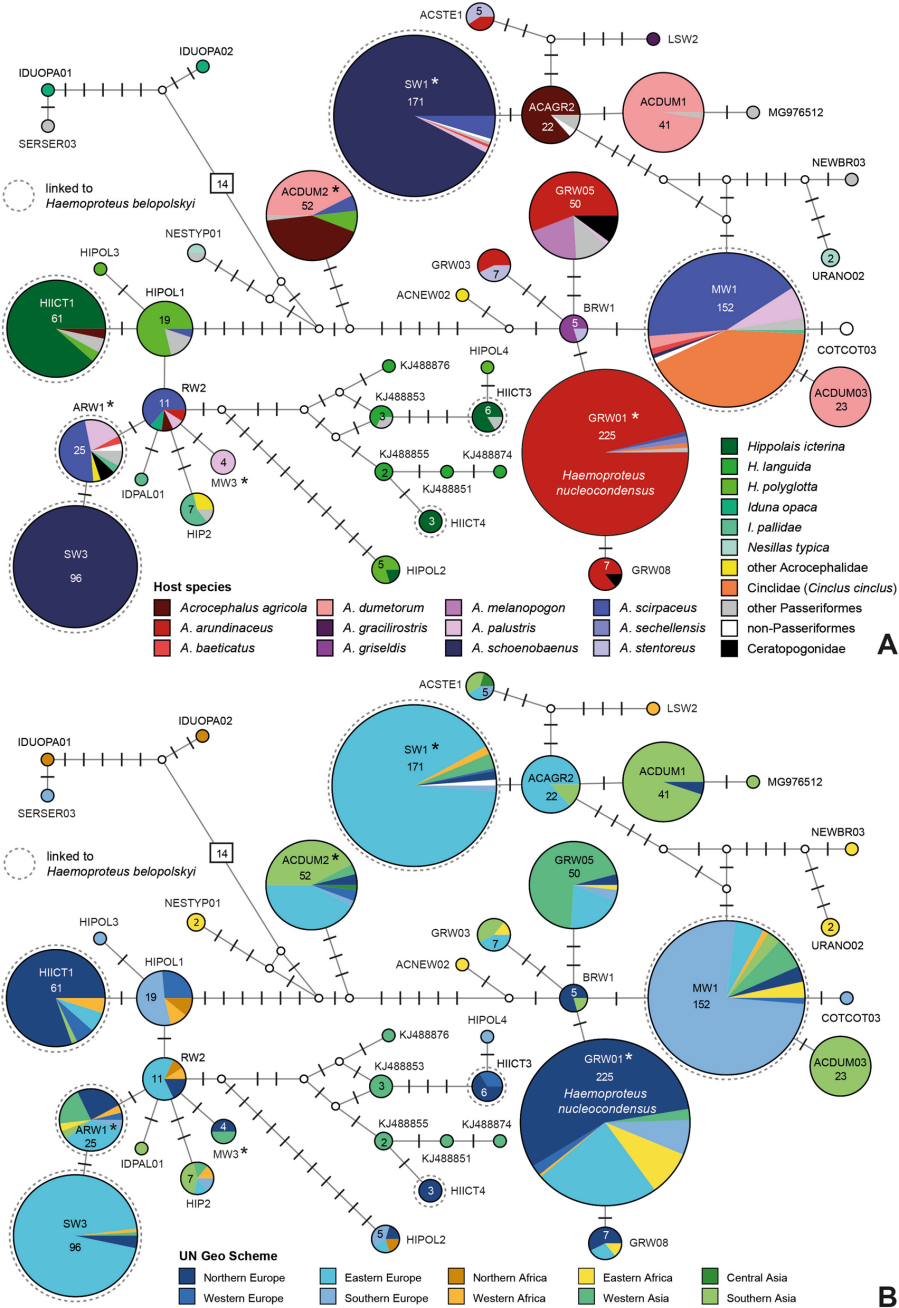
Median‑Joining DNA haplotype network of partial (474 bp) *CytB* sequences belonging to the *Haemoproteus belopolskyi* group. The two figures show the distribution in **A** bird families and **B** geographic areas according to the United Nations geoscheme. Each circle represents a unique haplotype/lineage. The frequency is indicated for all haplotypes with more than one record and roughly corresponds to the size of circles. Bars on branches and numbers in squares indicate the number of substitutions between two haplotypes. Small white circles represent median vectors, which are hypothetical (often ancestral or unsampled) sequences required to connect existing haplotypes with maximum parsimony. The lineages analysed in the present study are marked with asterisks

**Fig. 3:**
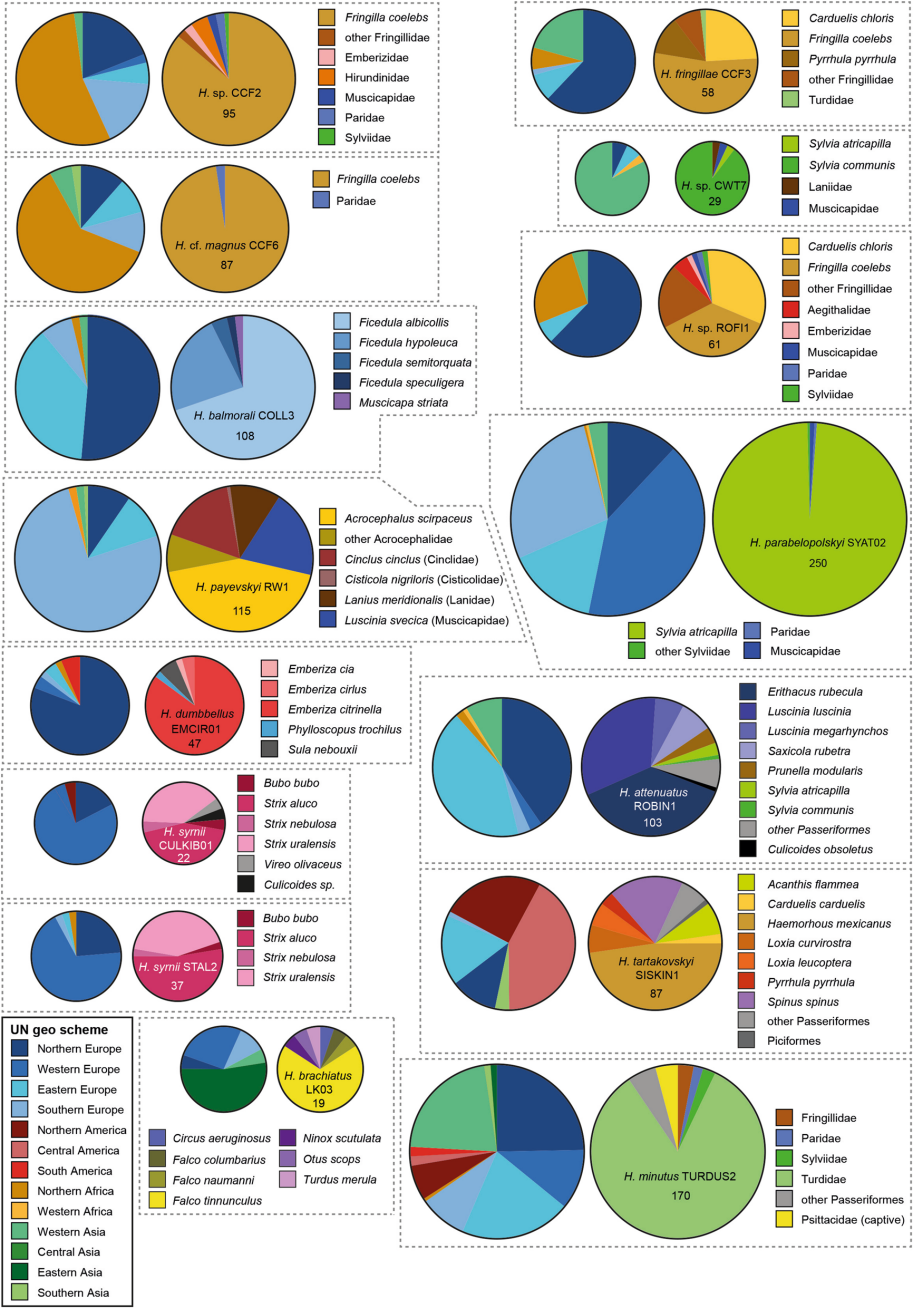
Pie charts showing the distribution of parasite lineages in geographic areas according to the United Nations geoscheme (left) and bird hosts (right)

The *H. belopolskyi* clade included 40 unique lineages differing by up to 38 bp in the 474 bp *CytB* sequence (Fig. 2). Hence, the distances between some lineages are much higher than in the *H. majoris* clade. They were almost exclusively found in various species of the family Acrocephalidae (marsh- and tree-warblers) in Europe, Asia, and Africa. According to the MalAvi database (http://130.235.244.92/Malavi/), the following nine lineages belong to *H. belopolskyi*: hARW1, hHIICT1, hHIICT3, hHIICT4, hHIICT5, hMW1, hRW3, hSW1, and hSW3. However, only HIICT1 and hMW1 were studied morphologically [30], and hHIICT5 (1 bp difference from hHIICT3) does not cover the entire barcode region. The clade also contains the lineage hGRW01, which was linked to *H. nucleocondensus* by [31], but differs only in 2 bp from *H. belopolskyi* hMW1. Further taxonomic studies might reveal that the lineages in the *H. belopolskyi* clade belong to multiple cryptic species. *18S* sequences were obtained from the following five lineages: hACDUM2, hARW1, hGRW01, hMW3, and hSW1. hACDUM2 (52 records) was found mainly in *Acrocephalus agricola* in Eastern Europe (22) and *Acrocephalus dumetorum* in Southern Asia (22). hARW1 (25) was found in *Acrocephalus scirpaceus* (12), *Acrocephalus palustris* (5), and a few other bird species in Northern, Western, and Eastern Europe, Western Asia, and Northern and Eastern Africa. hGRW01 (225) was almost exclusively detected in *Acrocephalus arundinaceus* (216) throughout its distribution range in Europe (195) and Africa (18), and Western Asia (3). hMW3 (6) was so far only found *in A. palustris* in Turkey (3) and in the present study in Lithuania (2). hSW1 (171) was mainly detected in *Acrocephalus schoenobaenus* (158) in Eastern Europe (152), Northern Europe (2), Western Europe (1), Western Asia (2), and Western Africa (1), and in *A. scirpaceus* (8) in Eastern Europe (3), Southern Europe (2), Western Asia (3), and Western Africa (1). The distributions of the other lineages (hCCF2, hCCF3, hCCF6, hCOLL3, hCWT7, hEMCIR01, hCULKIB01, hLK03, hROBIN1, hROFI1, hRW1, hSISKIN1, hSTAL2, hTURDUS2, and hSYAT02) in bird hosts and geographic areas (UN geoscheme) are shown as pie charts (Fig. 3). *Haemoproteus* sp. hCCF2 (95 records) was mainly found in *Fringilla coelebs* (81) in Northern Africa (52), Northern Europe (18), and Southern Europe (16). *Haemoproteus fringillae* hCCF3 (58) was detected mostly in *F. coelebs* (31), *Carduelis chloris* (14), and *Pyrrhula pyrrhula* (7) in Northern Europe (36), Western Asia (12), Eastern Europe (5), Northern Africa (4), and Southern Europe (1). *Haemoproteus* cf. *magnus* hCCF6 (87) was almost exclusively found in *F. coelebs* (85) in Northern Africa (53), Europe (27), Western Asia (5), and Southern Asia (2). *Haemoproteus balmorali* hCOLL3 (108) was detected in *Ficedula albicollis* (76), *F. hypoleuca* (24), and other Muscicapidae (9) in Northern Europe (56), Eastern Europe (41), Southern Europe (7), Western Asia (2), and Northern Africa (1). *Haemoproteus* sp. hCWT7 (29) was mostly found in *S. communis* (26) in Western Asia (24). *Haemoproteus syrnii* hCULKIB01 (22) was found in the owl species *Strix aluco* (10), *Strix uralensis* (9), *Bubo bubo* (1), and *Strix nebulosa* (1) in Europe. *Haemoproteus dumbbellus* hEMCIR01 (47) was almost exclusively found in *Emberiza citrinella* (40) in Europe. *Haemoproteus brachiatus* hLK03 (19) was mainly found in *Falco tinnunculus* (13) and other falcons (2) in Eastern Asia (8), Western Asia (1), and Europe (6). *Haemoproteus* sp. hROFI1 (61) was mainly found in *F. coelebs* (22), *Carduelis chloris* (20), and other Fringillidae species (12) in Northern Europe (38), Northern Africa (16), Eastern Europe (4), and Western Asia (3). *Haemoproteus attenuatus* hROBIN1 (103) was mostly found in the Muscicapidae species *Erithacus rubecula* (40), *Luscinia luscinia* (34), *Luscinia megarhynchos* (7), and *Saxicola rubetra* (7) in Europe and Western Asia. *Haemoproteus* sp. hRW1 (115) was detected in *A. scirpaceus* (62) and other Acrocephalidae (4), in *Cinclus cinclus* (24), *Lanius meridionalis* (16), *Luscinia svecica* (8), and *Cisticola nigriloris* (1) in Southern Europe (87), Eastern Europe (12), Northern Europe (11), and other regions (5). *Haemoproteus tartakovskyi* hSISKIN1 (87) was mostly found in Fringillidae (83) in Central America (37), Northern America (22), and Europe (26). The most common hosts were *Haemorhous mexicanus* (42), *Acanthis flammea* (7), *Loxia curvirostra* (6), *Loxia leucoptera* (5) in the Americas and *Spinus spinus* (16) in Europe. *Haemoproteus parabelopolskyi* hSYAT02 (250) was almost exclusively found in *S. atricapilla* (246) in Western Europe (103), Southern Europe (69), Eastern Europe (38), Northern Europe (30), Western Asia (8), and Africa (2). *Haemoproteus syrnii* hSTAL2 (37) was detected in owls in Europe (36) and Northern Africa (1), mainly in *Strix aluco* (19) and *Strix uralensis* (15). *Haemoproteus minutus* hTURDUS2 (170) was found mainly in Turdidae (140) in Europe (92), Western Asia (37), and the Americas (14). Most records originate from *Turdus merula* (121).

The information shown in the DNA haplotype networks and pie charts is summarised in Additional file 1: Table S1.

### *18S* sequence analyses

Molecular cloning was required in most cases to retrieve the *18S* sequences because the variants differed in their lengths. Only the samples AH1895H (hSYAT02) and AH1982H (hCOLL3) were not cloned because their *18S* sequences were fully readable following direct sequencing. Almost the complete *18S* sequences were cloned from 20 samples featuring 17 *Haemoproteus* lineages. Two to 15 clones were sequenced per sample (201 in total, 10.1 clones in mean). We intended to sequence up to 15 clones per lineage, but the yield of clones was extremely low in some cases. Of the 201 clones, 13 were excluded from the analyses because they originated from co-infections, which were not detected when sequencing the *CytB* barcode section with the standard primers by [12]. However, in some cases co-infections were visible either in the 885 bp sequences obtained with the primers CytB_HPL_intF1 and CytB_HPL_intR1 by [8] or the *18S* clones. Mostly the *18S* clones could be clearly assigned to one of the MalAvi lineages because other samples had single infections with the same lineages. The sample AH2168H featured a mixed infection with the lineages hCCF3 (6 clones), hCCF6 (7 clones), and hCCF5 (2 clones) of which only the hCCF3 clones were included in the analyses; the hCCF6 clones were excluded because samples AH2153H and AH2154H contained single infections with the same lineage, and the two hCCF5 clones were excluded because the mid parts of the sequences were unreadable. The sample AH1973H featured a co-infection with lineages hCCF3 (9 clones), hCCF5 (2 clones), and hROFI1 (1 clone). The hROFI1 clone was excluded from the analyses because the sample AH2171H provided sufficient clones of lineage hROFI1, but the two hCCF5 clones were kept because they were the only complete *18S* sequences of this lineage. The sample AH2171H contained a co-infection with hROFI1 (12 clones) and hCCF6 (3 clones); the hCCF6 clones were excluded from the analyses because sufficient hCCF6 clones were available from samples AH2153H and AH2154H. Moreover, sample AH1664H likely contained a co-infection with hSW1 and another *H. belopolskyi* lineage. The sample featured four clones (3, 5, 6, 14) closely resembling those of *H. belopolskyi* hARW1 (AH1903H) and hMW3 (AH1899 and AH1902), and seven clones (2, 4, 7, 8, 10, 11, 12) forming a separate branch within the *H. belopolskyi* clade. The first four clones might belong to lineage hSW3, which was exclusively found in the same host species (*A. schoenobaenus*) and differs only in one bp from hARW1 (Fig. 2). Double peaks in the electropherograms of the 885 bp *CytB* fragment matched lineage hSW3 but were too faint to clearly confirm its presence. Apart from AH1973H, AH2168H, AH2171H, and AH1664H, the other samples most likely contained mono-infections with *18S* clones belonging to single parasite lineages. The *18S* sequences of *H. majoris* hEMSPO03 (AH2023H) could not be fully retrieved because it featured poly-A and poly-T motives from position 1660 to 1800. The *18S* sequences were uploaded to NCBI GenBank under the accession numbers OR337936–OR338136. The 885 bp sections of the mitochondrial *CytB* were deposited under the accession numbers OR283176–OR283196.

**Fig. 4:**
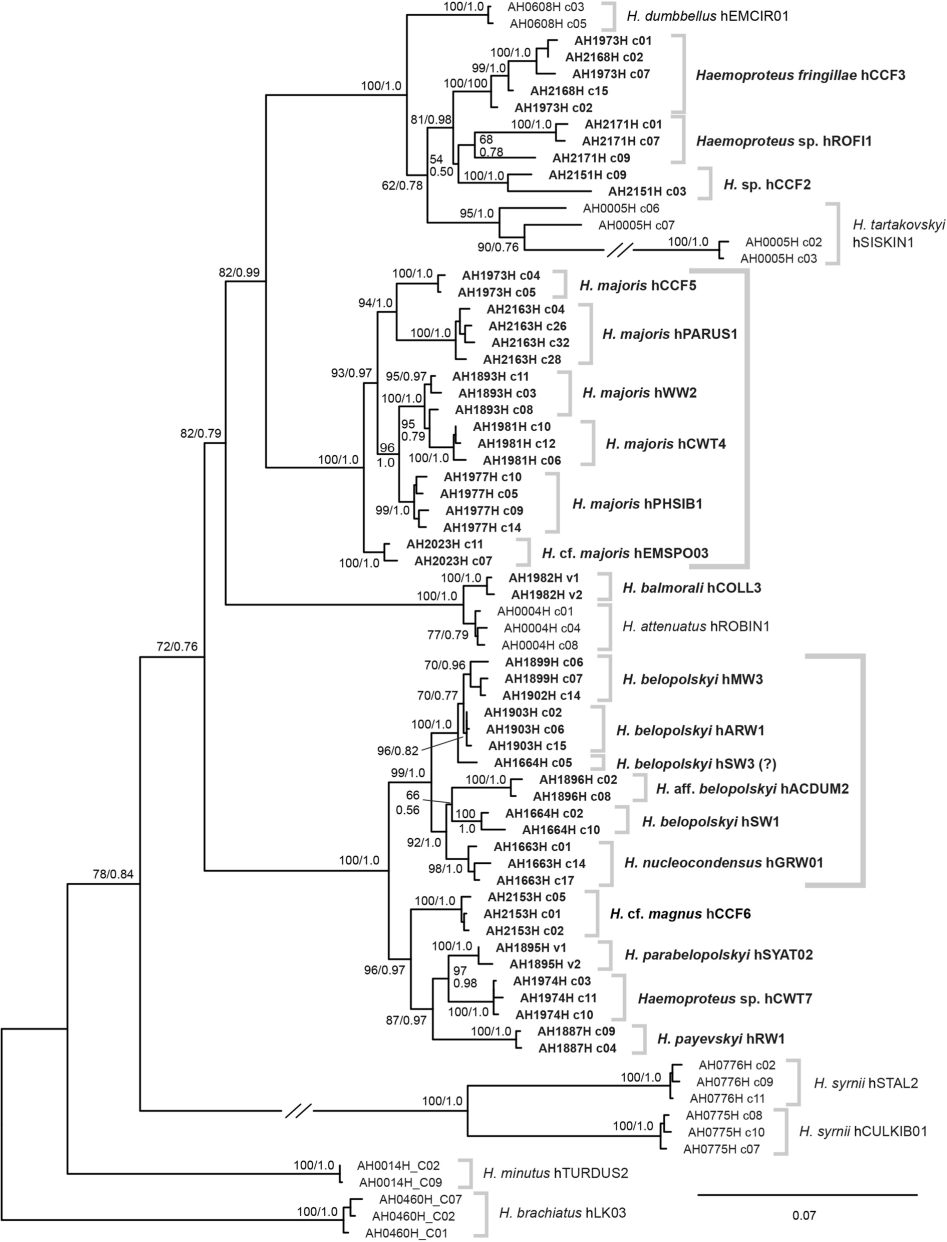
Bayesian inference tree of *Haemoproteus 18S* sequences. Posterior probabilities and maximum likelihood bootstrap values are indicated at most nodes. The scale bar indicates the expected mean number of substitutions per site according to the model of sequence evolution applied. The tree was midpoint-rooted, no outgroup was used

**Fig. 5:**
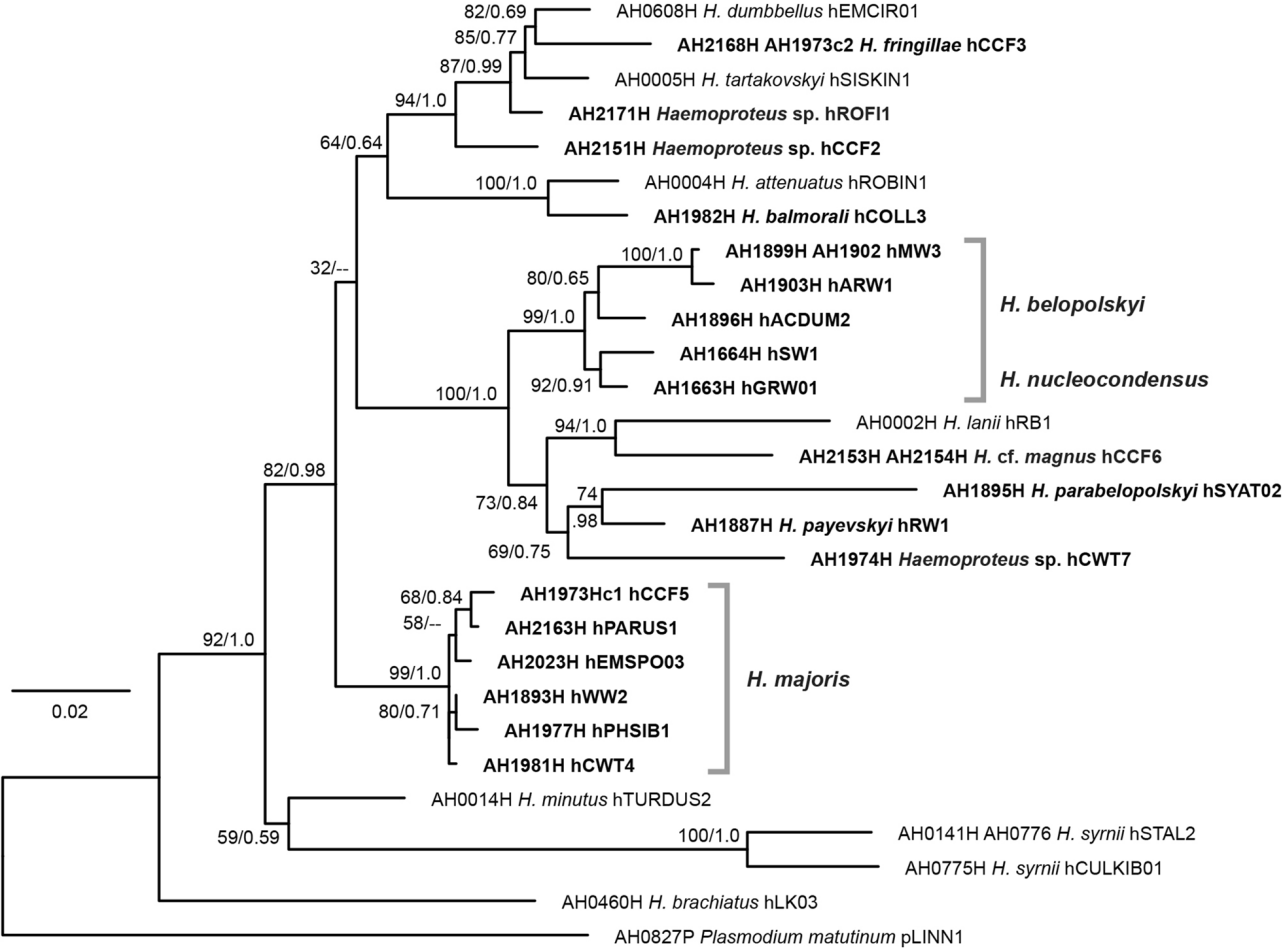
Bayesian inference tree of *Haemoproteus CytB* sequences (885 bp). Posterior probabilities and maximum likelihood bootstrap values are indicated at most nodes. The scale bar indicates the expected mean number of substitutions per site according to the model of sequence evolution applied. The tree was rooted with a sequence of *Plasmodium matutinum* pLINN1

Phylogenetic trees were calculated with the *18S* and *CytB* sequences of the present study and those published by [8]. The *18S* tree (Fig. 4), containing a selection of two to five distinct *18S* clones per MalAvi lineage (74 sequences in total), was mid-point rooted. The *CytB* tree (Fig. 5) was calculated with the 885 bp sequences and rooted with a sequence of *Plasmodium matutinum* pLINN1. The deeper nodes obtained low support values in both trees and the topology differed partially, but the *18S* and *CytB* trees shared some common patterns. The lineages *H. brachiatus* hLK03, *H. minutus* hTURDUS2, *H. syrnii* hCULKIB01, and *H. syrnii* hSTAL2 take more basal positions in the trees, whereas the other lineages cluster together in one clade with low (*18S*; bs/bp = 72/0.76) and moderate support (*CytB*; bs/pp = 82/0.98). The latter clade featured four highly supported subclades in both trees. The first subclade includes *H. dumbbellus* hEMCIR01, *H. fringillae* hCCF3, *Haemoproteus* sp. hROFI1, *H. tartakovskyi* hSISKIN1, and *Haemoproteus* sp. hCCF2. The second subclade includes the *H. balmorali* hCOLL3 and *H. attenuatus* hROBIN1. The third subclade features the *H. majoris* lineages hCCF5, hPARUS1, hWW2, hCWT4, hPHSIB1, and hEMSPO03 (not yet linked to a morphospecies). The fourth subclade includes the group of four *H. belopolskyi* lineages hMW3, hARW1, hSW1, hACDUM2, *H. nucleocondensus* hGRW01, and the lineages *H. parabelopolskyi* hSYAT02, *H. payevskyi* hRW1, *H.* cf. *magnus* hCCF6, and *H*. sp. hCWT7. The *18S* clones of most lineages clustered into reciprocally monophyletic clades. However, as mentioned above, the clones of sample AH1664H probably belong to two different *Haemoproteus* lineages (hSW1 and hSW3).

The *p*-distances between the *18S* variants of the 27 *Parahaemoproteus* lineages ranged from 0.46% in *H. lanii* hRB1 to 20.54% in *H. tartakovskyi* hSISKIN1 (Table 2). The latter lineage is exceptional because it featured two similar *18S* variants and a highly diverged third one. Excluding this sample, the mean maximum *p*-distance between *18S* clones of the other lineages was 1.84%. As mentioned above, some clones of sample AH1664H might belong to hSW3 (not confirmed) in co-infection. When testing the two sequence clusters separately, maximum *p*-distances between clones of hSW1 and hSW3 were less with 1.26 and 0.73, respectively (Table 2).

**Table 2:** Sequence features of the *Haemoproteus 18S* rRNA genes

ID	Species	Lineage	No. clones	GC-content (%)	Minimum length *18S*	Maximum length *18S*	Max. p-distance between variants	Recom-bination
AH0004H	*H. attenuatus*	hROBIN1	10	45.0	2129	2130	0.84	No
**AH1982H**	*H. balmorali*	hCOLL3	2	45.5	2116	2116	0.65	–
**AH1896H**	*H.* aff. *belopolskyi*	hACDUM2	7	46.0	2158	2159	0.82	No
**AH1899H**	*H. belopolskyi*	hMW3	2	45.7	2148	2148	1.36	–
**AH1902H**	*H. belopolskyi*	hMW3	3	45.7	2148	2148	0	No
**AH1664H**	*H. belopolskyi*	hSW1	11	45.9	2147	2150	5.64	No
**AH1903H**	*H. belopolskyi*	hARW1	13	45.8	2147	2147	0.97	No
AH0460H	*H. brachiatus*	hLK03	9	46.7	2007	2008	1.38	No
AH0608H	*H. dumbbellus*	hEMCIR01	6	44.3	2113	2114	0.58	No
**AH1973H**	*H. fringillae*	hCCF3	9	45.6	2027	2067	2.72	Yes
**AH2168H**	*H. fringillae*	hCCF3	6	45.7	2026	2061	2.98	Yes
AH0002H	*H. lanii*	hRB1	10	43.9	2017	2017	0.46	No
**AH1973H**	*H. majoris*	hCCF5	2	42.1	2278	2278	0.74	No
**AH1981H**	*H. majoris*	hCWT4	10	46.1	2144	2147	1.67	Yes
**AH2023H**	*H.* cf. *majoris*	hEMSPO03	9	42.8	2171	2176	0.72	No
**AH2163H**	*H. majoris*	hPARUS1	11	43.8	2181	2192	2.96	Yes
**AH1977H**	*H. majoris*	hPHSIB1	11	46.3	2153	2162	2.51	Yes
**AH1893H**	*H. majoris*	hWW2	9	45.6	2141	2149	1.99	Yes
**AH2171H**	*Haemoproteus* sp.	hROFI1	12	44.6	2063	2074	7.52	Yes
AH0014H	*H. minutus*	hTURDUS2	8	45.1	1943	1943	0.54	No
**AH1663H**	*H. nucleocondensus*	hGRW01	12	45.7	2158	2159	2.22	Yes
**AH1895H**	*H. parabelopolskyi*	hSYAT02	2	46.8	2161	2161	1.46	–
**AH1887H**	*H. payevskyi*	hRW1	13	44.8	2158	2160	0.96	No
**AH2151H**	*Haemoproteus* sp.	hCCF2	13	44.7	2096	2105	3.43	No
**AH2153H**	*H.* cf. *magnus*	hCCF6	12	45.3	2163	2163	0.97	No
**AH2154H**	*H.* cf. *magnus*	hCCF6	5	45.3	2163	2163	1.86	No
**AH1974H**	*Haemoproteus* sp.	hCWT7	14	46.2	2159	2160	1.16	No
AH0775H	*H. syrnii*	hCULKIB01	9	45.6	2177	2182	1.97	Yes
AH0141H	*H. syrnii*	hSTAL2	8	47.4	2141	2147	1.19	Yes
AH0776H	*H. syrnii*	hSTAL2	11	47.4	2141	2146	1.20	Yes
AH0005H	*H. tartakovskyi*	hSISKIN1	10	39.4	2046	2099	20.54	Yes

The approximate total lengths of the *18S* sequences (including missing parts and primer regions) ranged from 1943 bp in *H. minutus* hTURDUS2 to 2278 bp in *H. majoris* hCCF5. The largest differences between the shortest and longest *18S* sequences were found in *H. tartakovskyi* hSISKIN1 with 53 bp and in *H. fringillae* hCCF3 with 40 bp; the mean difference over all 27 *Parahaemoproteus* lineages was 6.8 bp. The GC contents ranged from 39.4% in *H. tartakovskyi* hSISKIN1 to 47.4% in *H. syrnii* hSTAL2; the overall mean was 45.2% (Table 2). Interestingly, *H. tartakovskyi* hSISKIN1 featured three *18S* clones with 42.7% (short branches in Fig. 4) and seven clones with 37.4% GC-content (long branch in Fig. 4).

Tests for recombinant signals were performed separately for the alignments of *18S* clones from each *Parahaemoproteus* lineage using RDP5 [19]. Recombinant signals were detected in ten of the 27 lineages investigated, including *H. fringillae* hCCF3, *Haemoproteus* sp. hROFI1, *H. nucleocondensus* hGRW01, *H. syrnii* hCULKIB01, *H. syrnii* hSTAL2, *H. tartakovskyi* hSISKIN1, and the *H. majoris* lineages hCWT4, hPHSIB1, hPARUS1, and hWW2 (Table 2). The strongest recombination signals were detected in hPHSIB1, hWW2, and hCCF3 with each five or more of the seven tests providing significant results (Additional file 2: Table S2).

### Probes for in situ hybridization

Based on the alignment containing all *Haemoproteus 18S* sequences available, oligonucleotide probes were designed to specifically target the *18S* rRNA of the lineages investigated in this study. The *18S* sequences of most *Haemoproteus* lineages featured unique sequence regions, which could be targeted with oligonucleotide probes by in situ hybridization (Table 3). However, *18S* sequences of the *H. belopolskyi* lineages hARW1, hMW3, and hSW3 were too similar and a probe targeting all three lineages in parallel was designed. The probe designed for lineage hROFI1 only targets one of the two main variants (C02, C08, C09, C11); the probe was already tested, confirming the expression in the bird hosts (unpublished results). The lengths of the probes and the annealing temperatures ranged from 23 to 31 nucleotides and 54.2 °C and 68.1 °C, respectively. Most probes obtained a maximum (100) quality score in AmplifX v.2.0.7.

**Table 3:** In silico tested probes for in situ hybridization

Species	MalAvi lineage	Probe name	Probe sequence (5′–3′)	Length (bp)	Tm (°C)
*H. attenuatus*	hROBIN1	hROBIN1-18S	CTCGCAATTTAGCCGAAACTAAACTACAAG	30	63.3
*H. balmorali*	hCOLL3	hCOLL3-18S	TCCCCTTCCTTGCAGAACAAGAAAGA	26	64.3
*H. belopolskyi*	hACDUM2	hACDUM2-18S	GGATATATTTTCCAGGTACGCAAAC	25	58.3
*H. belopolskyi*	hARW1, hMW3, hSW3*	hARW1_hMW3_SW3-18S	AGGGCGAACCTCACTGTCTAAAGC	24	64.7
*H. belopolskyi*	hSW1	hSW1-18S	CAAAGACGAGCTTCGCTATTTTAAGCC	27	63.2
*H. brachiatus*	hLK03	hLK03-18S	TGTCAATCTACACCGTCTAGCCA	23	61.1
*H. dumbbellus*	hEMCIR01	hEMCIR01-18S	CAACCTCCCTTTAATTATAGCATCCGCGAAG	31	65.7
*H. fringillae*	hCCF3	hCCF3-18S	CACACTCCACTAATCGAGTTTATACCTTCCG	31	64.8
*H. lanii*	hRB1	hRB1-18S	CTCTACGCGTAATAAATTACGGCA	24	59.3
*H.* cf. *magnus*	hCCF6	hCCF6-18S	ACGGCAAAGAAACTTCGCTATTTCAAGTC	29	64.7
*H. majoris*	hCCF5	hCCF5-18S	CGCCCTAGTTTTACAAAAACAAATCTC	27	59.9
*H. majoris*	hCWT4	hCWT4-18S	TCCTCACAAACTGGATCGATGCCA	24	64.2
*H.* cf. *majoris*	hEMSPO03	hEMSPO03-18S	TCGTCGTAGTTACGCACAAACTATCC	26	63.0
*H. majoris*	hPARUS1	hPARUS1-18S	CGAATACGCCACCCGAAAGTGACAACAAG	29	68.1
*H. majoris*	hPHSIB1	hPHSIB1-18S	CTACTATAAGCGATATCGACGTAGT	25	57.6
*H. majoris*	hWW2	hWW2-18S	ACAAATCCTCTCTTTAGGTAAAATGGCA	28	61.2
*H. minutus*	hTURDUS2	hTURDUS2-18S	CTCCATGTTACCAGTAAAGACTCTCA	26	60.1
*H. nucleocondensus*	hGRW01	hGRW01-18S	GGCTAAGACAAGCAAAGCTATCT	23	58.8
*H. parabelopolskyi*	hSYAT02	hSYAT02-18S	CAGAACTTTAAGCGGAACAGCACTGTGC	28	66.8
*H. payevskyi*	hRW1	hRW1-18S	GCAGCCTTCAGATAACTGTAAAAAGCTATCG	31	64.4
*H. syrnii*	hCULKIB01	hCULKIB01-18S	CAACCGATTCAAACCTTCGTTGCC	24	63.4
*H. syrnii*	hSTAL2	hSTAL2-18S	ACTTCCCGAAGAAAGCTGGATATCC	25	62.4
*H. tartakovskyi*	hSISKIN1	hSISKIN1-18S	CTAGCCTCGGGCGATGTTCTCCAAG	25	66.9
*Haemoproteus* sp.	hCCF2	hCCF2-18S	AAATAGGAAAGCGATGCAGGCAA	23	61.4
*Haemoproteus* sp.	hCWT7	hCWT7-18S	CACCATGCGTGAACACAATGCAGC	24	54.2
*Haemoproteus* sp.	hROFI1	hROFI1-18S	TTCCGCGACAGCCATAACAACCACC	25	65.6

*The presence of hSW3 *18S* clones in sample AH1664 could not be confirmed with certainty

## Discussion

For the present study, the *18S* rRNA genes of 19 *Haemoproteus* lineages, all belonging to the subgenus *Parahaemoproteus*, were sequenced. More than half of these lineages were previously linked to or are closely related to the *H. majoris* (hCCF5, hCWT4, hEMSPO03, hPARUS1, hPHSIB1, hWW2) and *H. belopolskyi* (hACDUM2, hARW1, hMW3, hGRW01, hSW1) species groups based on morphological characters of their blood stages and/or similar *CytB* barcode sequences. The two species groups include some of the most common *Haemoproteus* lineages found in songbirds of the Palearctic region. Moreover, the *18S* rRNA genes of eight other *Haemoproteus* lineages were sequenced: hCOLL3, hCCF2, hCCF3, hCCF6, hCWT7, hROFI1, hRW1, and hSYAT02. The data on eight *Haemoproteus* lineages published by [8], hCULKIB01, hEMCIR01, hLK03, hROBIN1, hRB1, hSISKIN1, hSTAL2, and hTURDUS2, were included in the sequence analyses.

Most mammalian *Plasmodium* species feature two highly diverged sequence clusters, each containing one or two similar *18S* variants. The *18S* variants of *Plasmodium falciparum*, *Plasmodium vivax*, and *Plasmodium berghei* were shown to be differentially expressed in the vertebrate hosts (A-type) and mosquito vectors (S-type) [32–34]. *Plasmodium vivax* is the only species with an additional O-type variant, which is expressed in the ookinetes and oocysts [33]. Another exception is *Plasmodium malariae* because its *18S* genes are almost identical and do not form separate clusters. The distances between *18S* variants were also comparably high in *Plasmodium vaughani* pSYAT05 (9.3%), *Plasmodium matutinum* pLINN1 (10.8%), and *Plasmodium elongatum* pGRW06 (14.9%) [8]. Recombination tests detected chimeric features in the *18S* sequences of several *Plasmodium* species, suggesting that distinct variants do not evolve independently according to a model of birth-and-death evolution under strong purifying selection [4] but rather in a semi-concerted fashion [8, 35].

The *Haemoproteus* parasites studied also featured distinct *18S* variants, but the averaged maximum distances between the variants were considerably lower (mean of 1.84% excluding the aberrant *H. tartakovskyi* hSISKIN1) than in most *Plasmodium* species investigated. Unlike in many *Plasmodium* species, the *18S* variants of most lineages clustered into reciprocally monophyletic clades. An exception was sample AH1664H, whose *18S* variants differed by 5.64% and fell into separate clades within the *H. belopolskyi* group, however, the latter sample likely featured a co-infection with *H. belopolskyi* hSW3 and *H. belopolskyi* hSW1 (see “Results”). *Haemoproteus* sp. hROFI1 featured two *18S* variants diverged by 7.5%, but they clustered together in a weakly supported clade (Fig. 4); apart from double peaks matching hCCF6 in the long *CytB* sequences, no other lineage in co-infection was identified. The most exceptional patterns were found in *H. tartakovskyi* hSISKIN1, which featured three *18S* variants, two similar ones separated by 6.5% and a third one separated from the latter two by 18.3% and 20.5%, respectively. The third variant showed a high number of substitutions (mostly changes G/C to A/T) in sequence regions, which were conserved in most other *Haemoproteus* lineages, therefore its GC-content was considerably lower with 37.4% compared to that of the other variants with 43.2% and 42.4%. This aberrant *18S* variant might be non-functional and therefore acquired a much higher number of substitutions compared to other copies of the *Haemoproteus 18S* rRNA genes. Despite the high sequence divergence, all three variants clustered in a well-supported clade (Fig. 4). The nuclear genome sequences of *H. tartakovskyi* hSISKIN1 published by Bensch et al*.* [36] also included the aberrant third variant (PRJNA309868, contig 65) but only one of the other two (PRJNA309868, contig 602). The mean GC-content of all *Haemoproteus 18S* sequences analysed was 45.2%, which is considerably higher than that of avian *Plasmodium* spp. with 34.0% and *common Leucocytozoon* spp. with 37.3%. The GC-content was only higher in parasite lineages belonging to the *Leucocytozoon toddi* species group with 49.3% [8]. The approximate total lengths of the *Haemoproteus 18S* sequences ranged from 1943 bp in *H. minutus* hTURDUS2 to 2278 bp in *H. majoris* hCCF5 and, therefore, was more variable than in avian *Plasmodium* spp. (2100 to 2177 bp), *Leucocytozoon* spp. (2094 to 2138 bp), and the *Leucocytozoon toddi* species group (2125 to 2308 bp). The recombination tests conducted with the *18S* sequences of the *Haemoproteus* parasite lineages also indicated chimeric features potentially resulting from recombination between *18S* variants of the same species or lineages. The results differed between species, e.g., no recombinant *18S* variants were detected in the *H. belopolskyi* group compared to the *H. majoris* group with four out of five lineages featuring recombinant variants. Thus, the results suggest that the nuclear ribosomal genes of *Haemoproteus* species rather evolve in a semi-concerted fashion as suggested for several *Plasmodium* species by Corredor and Enea [35] than according to a model of birth-and-death evolution as proposed by Rooney [4]. Corredor and Enea [35] used the term semi-concerted evolution because they found that some (but not all) *18S* rRNA gene copies of *Plasmodium* spp. evolve in concert, thus requiring some form of sequence interaction (conversion) other than unequal crossing over. In contrast, the ribosomal genes of most eukaryotes evolve in a fully concerted fashion, leading to the homogenization of individual gene copies [3] involving mechanisms such as unequal crossing over during recombination, gene duplication, and inter-chromosomal gene conversion [1, 2, 37]. The model of birth-and-death evolution on the other hand assumes that multigene families involved in the immune system, such as immunoglobulins and the major histocompatibility complex (MHC), do not evolve in a concerted fashion; new copies originate by gene duplication, whereas others become non-functional and deleted over time [38, 39].

The *18S* rRNA gene has been used as the standard reference sequence when screening for apicomplexan parasites and determining their phylogenetic relationships. However, due to the presence of distinct variants and recombination between them, long inserts/deletions, and GC-contents strongly varying between genera/subgenera, the *18S* sequences of haemosporidian parasites are less suitable as a phylogenetic marker than in other groups of apicomplexan parasites (e.g., Eucoccidiorida, Piroplasmorida, and Eugregarinorida), which mostly possess identical and more conserved copies of nuclear ribosomal genes. Therefore, PCR screening assays for human *Plasmodium* species only target one of the main *18S* variants [40, 41]. More recent approaches even established quantitative reverse transcription PCR (qRT-PCR) to directly target the *18S* rRNA, resulting in even higher sensitivity [42, 43]. These approaches are practical when screening for human *Plasmodium* infections because they comprise only four species, but less suitable when screening for haemosporidian parasites of wild birds because they include a vastly higher number of species and obtaining their *18S* sequences would require molecular cloning or the use of next generation sequencing methods.

However, the *18S* and other nuclear ribosomal RNA genes, respectively the corresponding ribosomal RNAs, are extremely useful targets for in situ hybridization assays to label parasites in the host tissue. That opens new perspectives for pathology research by targeting certain parasite species/lineages during avian haemoproteosis, which can markedly damage various bird organs but remains insufficiently investigated [44, 45]. One of the main advantages compared to other target sequences is that each cell contains numerous ribosomes, resulting in a higher sensitivity. Moreover, ribosomal RNAs are considerably more stable than other RNAs, which is particularly important when analyzing pathological samples that were not prepared from fresh material [46]. Genus-specific oligonucleotide probes for in situ hybridization have been established to target and differentiate between avian *Plasmodium*, *Haemoproteus*, and *Leucocytozoon* parasites in bird tissue [47, 48]. A *Plasmodium*-specific probe was successfully used to detect blood and tissue stages in paraffin-embedded organs of captive penguins and wild passeriform birds, showing that tissue stages of *Plasmodium* spp. can cause mortality in both groups [47, 49]. Chromogenic in situ hybridization assays have also been performed to characterise tissue meronts in accipitriform raptors [50], strigiform raptors [51], thrushes [52], and other songbirds [11]. The latter studies provided valuable information regarding the development of exo-erythrocytic parasite stages in host tissue. For example, the combination of histological methods and in situ hybridization led to the first report of megalomeronts in *Haemoproteus syrnii* hSTAL2 and the characterization of a new mode of exo-erythrocytic development in *Leucocytozoon* sp. lSTAL5 [51]. The new *Haemoproteus 18S* sequences and oligonucleotide probes could be used to specifically target certain parasite lineages/species in host tissue. This particularly important and remains the only available approach when samples contain co-infections, which predominate in wildlife.

## Conclusion

For the present study, the *18S* rRNA genes of 19 *Haemoproteus* lineages belonging to the subgenus *Parahaemoproteus,* the most common blood parasites of Palearctic birds, were sequenced, thereby focusing on the *H. belopolskyi* and *H. majoris* species groups. The *18S* sequences of eight additional *Haemoproteus* species, published previously by Harl et al*.* [8], were included in the analyses. To show the geographic and host distribution of the lineages investigated, DNA haplotype networks and pie charts were prepared based on the *CytB* data available in the MalAvi database. Like most *Plasmodium* parasites, the *Haemoproteus* lineages also featured two or more *18S* variants, but the intraspecific distances between variants of the same lineages were generally lower. Moreover, the *18S* sequences of all but one parasite lineage clustered into reciprocally monophyletic clades, which was not the case for most mammalian and avian *Plasmodium* species. The presence of chimeric features in the *18S* variants of more than one third of the *Haemoproteus* lineages indicates that their ribosomal units evolve in a semi-concerted fashion rather than according to a strict model of birth-and-death evolution. Based on an alignment of the *18S* sequences, oligonucleotide probes were designed in silico, which could be used to specifically target parasites species/lineages in the host tissue with chromogenic or fluorescent in situ hybridization methods. This would allow the detection of certain parasite lineages even in samples with co-infections, which are extremely common in songbirds.

### Supplementary Information

**Additional file 1: Table S1.** *CytB* sequence data used for the DNA haplotype networks and pie charts showing the distribution of *Haemoproteus* lineages in bird hosts and geographic regions.

**Additional file 2: Table S2.** Results of the RDP5 recombination tests analyzing the *18S* sequences of the *Haemoproteus* lineages studied.

## Data Availability

The *18S* and *CytB* sequence data were deposited in NCBI GenBank under the following accession numbers: OR337936–OR338136 (*18S*) and OR283176–OR283196 (*CytB*).

## Abbreviations

*18S*: *18**S* ribosomal RNA gene
*CytB*: *Cytochrome B* gene
SSU: small ribosomal subunit
LSU: large ribosomal subunit
AICc: Akaike Information Criterion corrected
aff.: affinis (the proposed species is related to but not identical to the species of this binomial name
here: The lineage is genetically closely related to the named species, but its morphology has not been studied yet)
cf.: conferatur (compare with the named species)
